# Implication of Trimethylamine N-Oxide (TMAO) in Disease: Potential Biomarker or New Therapeutic Target

**DOI:** 10.3390/nu10101398

**Published:** 2018-10-01

**Authors:** Manuel H. Janeiro, María J. Ramírez, Fermin I. Milagro, J. Alfredo Martínez, Maite Solas

**Affiliations:** 1 Department of Pharmacology and Toxicology, University of Navarra, 31008 Pamplona, Spain; mjaneiro@alumni.unav.es (M.H.J.); mariaja@unav.es (M.J.R.); 2 IdISNA, Navarra Institute for Health Research, 31008 Pamplona, Spain; 3 Department of Nutrition, Food Science and Physiology, Centre for Nutrition Research, University of Navarra, 31008 Pamplona, Spain; fmilagro@unav.es (F.I.M.); jalfmtz@unav.es (J.A.M.); 4 CIBERobn, CIBER Fisiopatología de Obesidad y Nutrición, Instituto de Salud Carlos III, 28029 Madrid, Spain; 5 IMDEA Food Institute, 28049 Madrid, Spain

**Keywords:** microbiota, gut dysbiosis, cardiovascular disease, neurological disorder, inflammation, antibiotics

## Abstract

Trimethylamine N-oxide (TMAO) is a molecule generated from choline, betaine, and carnitine via gut microbial metabolism. The plasma level of TMAO is determined by several factors including diet, gut microbial flora, drug administration and liver flavin monooxygenase activity. In humans, recent clinical studies evidence a positive correlation between elevated plasma levels of TMAO and an increased risk for major adverse cardiovascular events. A direct correlation between increased TMAO levels and neurological disorders has been also hypothesized. Several therapeutic strategies are being explored to reduce TMAO levels, including use of oral broad spectrum antibiotics, promoting the growth of bacteria that use TMAO as substrate and the development of target-specific molecules. Despite the accumulating evidence, it is questioned whether TMAO is the mediator of a bystander in the disease process. Thus, it is important to undertake studies to establish the role of TMAO in human health and disease. In this article, we reviewed dietary sources and metabolic pathways of TMAO, as well as screened the studies suggesting possible involvement of TMAO in the etiology of cardiovascular and neurological disorders, underlying the importance of TMAO mediating inflammatory processes. Finally, the potential utility of TMAO as therapeutic target is also analyzed.

## 1. Introduction

There is a growing awareness that intestinal microbial organisms, collectively termed microbiota, participate in the global metabolism of their host [1,2,3], including intestinal health, immune function and/or bioactivation of nutrients and vitamins. More recently microbiota of humans has been linked with complex disease phenotypes such as obesity and insulin resistance [4,5,6]. Also, the potential role of a complex phosphatidylcholine–choline metabolic pathway involving gut microbiota in contributing to the pathogenesis of atherosclerotic coronary artery disease in animal models and humans has been described [7]. Choline, a trimethylamine-containing compound and part of the head group of phosphatidylcholine, is metabolized by gut microbiota to produce an intermediate compound known as trimethylamine (TMA). TMA is rapidly further oxidized by hepatic flavin monooxygenases to form trimethylamine N-oxide (TMAO). TMAO was thought to be a waste product of choline metabolism without action in our organism but nowadays there is convincing evidence suggesting an association between TMAO and inflammation [8,9,10,11,12]. Furthermore, in the last decade the studies suggesting an association between high plasma TMAO levels and risk for developing atherosclerosis have increased markedly [7,13,14,15,16,17]. However, the exact mechanism underlying this correlation is still unknown.

### 1.1. TMAO Metabolism

**Figure 1:**
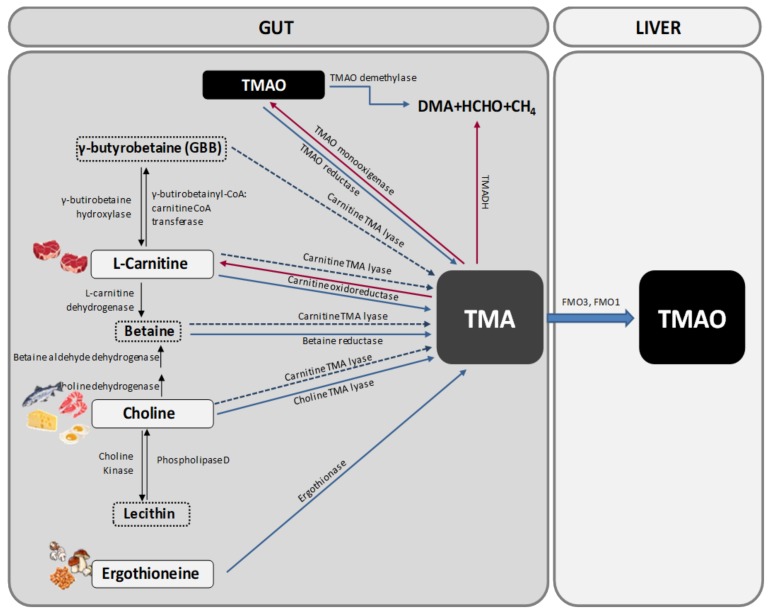
Pathways for trimethylamine N-oxide (TMAO) formation. Trimethylamine (TMA) is formed in the intestinal lumen when gut microbiota metabolize carnitine, choline, and choline-containing compounds in the diet. TMA can be absorbed from the intestine. This absorbed TMA is delivered to the liver where flavin-dependent monooxygenase (FMO) isoforms 1 and 3 convert it to TMAO. DMA: dimethylamine; HCHO: formaldhyde; TMADH: trimethylamine dehydrogenase; CoA: coenzyme A.

Trimethylamine N-oxide is generated from the oxidation of TMA that occurs in the gut microbiota. TMA is generated in the gut from betaine, L-carnitine and its metabolite γ-butyrobetaine (GBB), choline and other choline-containing compounds, which are present in the diet. These precursors are converted into TMA by various enzymes [18] (Figure 1).

Choline can be found in many foods, mainly in those of animal origin as free choline or as a part of several compounds (phosphatidylcholine, phosphocholine, sphingomyelin, etc.). One of the main choline-containing compounds is phosphatidylcholine, also known as lecithin, which can be converted into choline by the enzyme Phospholipase D. Interconversion of lecithin and choline is bidirectional, being the conversion of choline to lecithin catalysed by choline kinase [19] (Figure 1).

Choline is transformed into TMA by the action of the enzyme choline TMA lyase while betaine, which is found mostly in plants, is reduced to TMA by betaine reductase in a coupled reduction-oxidation reaction where it acts as an electron acceptor. Betaine also plays an important role as a methyl donor in the betaine homocysteine methyltransferase pathway. Alternatively, choline can be transformed into betaine by the sequential action of two enzymes (choline dehydrogenase and betaine aldehyde dehydrogenase) [19] (Figure 1).

Apart from choline, the other main precursor of TMA is L-carnitine. Carnitine oxidoreductase is the main enzyme responsible for the conversion of L-carnitine into TMA [20] (Figure 1). However, there is another enzyme called Carnitine TMA lyase with substrate promiscuity for carnitine, choline, betaine and GBB. Carnitine is only found in foods as its L-stereoisomer, with animal products containing the biggest amounts [18,19]. L-carnitine can also be transformed into two other precursors, i.e., betaine by the action of L-carnitine dehydrogenase and GBB by the enzyme γ-butyrobetainyl-CoA:carnitine CoA transferase. GBB can be reconverted into L-carnitine by the enzyme γ-butyrobetaine hydroxylase or be transformed into TMA by the action of the previous cited enzyme carnitine TMA lyase [19] (Figure 1).

Another source of TMA is ergothioneine, a biogenic amine (a derivative of histidine) that can be obtained from some dietary sources, such as mushrooms, some meat products (mainly liver and kidney), and several types of beans. The degradation of ergothioneine by the enzyme ergothionase produces TMA [19] (Figure 1).

Finally, TMAO and TMA can also be found in a natural way in some foods like fish. Approximately 50% of the ingested TMAO is absorbed unchanged and then excreted in urine. The remaining 50% is converted into TMA in the gut by the action of TMAO reductase [19]. TMA can be oxidized to TMAO again by the action of TMA monooxigenase, which is present in some gut microorganisms [21,22] (Figure 1). Moreover, some bacteria are able to deplete TMA and TMAO to form dimethylamine (DMA) and formaldehyde by the action of the enzymes trimethylamine dehydrogenase (TMADH) and TMAO demethylase [22] (Figure 1).

Most of TMA ingested or formed in the gut is rapidly absorbed into the portal circulation by passive diffusion and then oxidized to TMAO by the action of hepatic flavin containing monooxygenases FMO3 and FMO1 (Figure 1). FMO1 has tenfold lower specific activity in the liver than does FMO3, so FMO3 is the main enzyme responsible for the conversion of TMA into TMAO [18].

### 1.2. TMA and TMAO Distribution and Excretion

The metabolism of dietary TMAO in humans is poorly understood, although it has been purported to enter the same metabolic pathway as its putative precursors. To quantitatively elucidate the metabolic fate of orally consumed TMAO, a recent study traced the metabolic fate of orally consumed TMAO in humans using a stable isotope approach [23]. They reported that orally consumed TMAO is largely absorbed and does not require microbial or hepatic processing, has a high turnover and rapid clearance rate and is taken up by extrahepatic tissues.

Some of the TMA ingested and formed in the gut can be eliminated in feces, but the amount of TMA excreted in feces has not been carefully quantified. As mentioned before, most of TMA is absorbed by passive diffusion across the enterocyte membranes. Nearly 95% of TMA is oxidized and it is afterwards excreted in urine in a 3:95 TMA: TMAO ratio within 24 h, only 4% is excreted in feces and less than 1% is eliminated in the breath [18].

Trimethylamine N-oxide can also be metabolized to dimethylamine (DMA), formaldehyde, ammonia and methane by some methanogenic bacteria containing the enzyme TMAO demethylase [22].

### 1.3. TMAO Detection and Measurement

In most studies, TMAO is measured in urine and plasma samples although it is sometimes measured in serum samples too. The methods that are often used include liquid chromatography mass spectrometry (LC-MS), mainly stable isotope dilution high performance liquid chromatography with electrospray ionization tandem mass spectrometry (DIS-HPLC-SM/SM), proton nuclear magnetic resonance spectrometry (1H-NMR), headspace gas chromatography (GC) and matrix-assisted laser desorption/ionization time-of-flight mass spectrometry (MALDI-TOF-MS). Other technique less common is Fast Atom Bombardment- mass spectrometry (FAB-SM).

It has been recently described a new method to measure TMAO in urine combining TMA derivation with ethyl-bromoacetate and liquid chromatography with single ion monitoring (LC-SIM) [24].

Furthermore, in some studies TMAO has been measured in liver [25] and fecal waters [26] using 1H-NMR but it has also been measured in other tissues such as brain, muscle, kidney or intestine using the method of reduction from TMAO to TMA with a mixture of ferrous sulphate and EDTA as described by Wekell and Barnett (1991) [27] or a modification of their method [28,29]. Additionally, in a recent study, TMAO was detected and measured in cerebrospinal fluid using LC-MS [30].

### 1.4. Variations in TMAO Levels

Plasma TMAO levels show wide inter- and intra-individual variations. These levels are influenced by several factors [31]. Some studies performed in human and rats have revealed that plasma TMAO levels show an age related fashion, i.e., levels increase with age [32,33]. Another influencing factor is cholic acid, a bile salt that can induce FMO3 expression via the bile acid–activated nuclear receptor FXR, thereby increasing plasma TMAO levels. Other inductors of FMO3 are oestrogens, while testosterone acts as a suppressor. Additionally, TMAO levels decrease at the onset and during menstruation causing trimethylaminuria [18].

Diet also plays a key role in TMAO formation. For example, vegetables of the family *Brassicaceae* can reduce FMO3 activity. Moreover, vegetarians present a different gut microbiota in comparison to omnivorous people and they are less able to produce TMA from L-carnitine [14]. Furthermore, high fat diets or Western-like diets increase plasma TMAO levels [8,34,35,36,37,38,39] (Table 1). The amount of protein in the diet seems to have a high positive correlation with TMAO excreted in urine [40]. A possible explanation for this finding is that TMAO may be synthetized to arrange the excess of amine groups as it occurs in some marine animals [41]. Moreover, a diet low in proteins in patients with chronic kidney disease (CKD) resulted in lower plasma TMAO levels [42]. Additionally, some studies suggest that a diet high in non-digestible carbohydrates can reduce TMAO formation by remodelling gut microbiota [26] while other studies reports the opposite effect, suggesting that a diet high in non-digestible starch increases plasma TMAO levels in the short term [43] (Table 1).

Diets with pistachio supplementation seem to reduce TMAO formation [44] while histidine supplementation seems to increase plasma and urine TMAO levels [45]. Another study demonstrated that high plasma TMAO concentrations may reflect a specific metabolic pattern characterized by hypomethylation and low HDL and phospholipids [45,46] (Table 1).

Finally, renal clearance may play a major and critical role in plasma TMAO levels. Mueller et al., (2015) [47] did an 8 years follow-up study trying to relate plasma TMAO, betaine or choline levels with cardiovascular disease (CVD), but the results showed that the levels were confounded by impaired kidney function or poor metabolic control and they were not associated with presence, incidence or events of coronary heart disease. Other studies revealed that TMAO is augmented in renal insufficiency but concentrations normalize after renal transplantation [48] and levels are higher the more the glomerular filtration rate GFR is reduced [49]. However, there are also some studies suggesting that TMAO can contribute to the development of renal insufficiency [50] and/or serve as a predictor of glomerular injury [51].

## 2. Importance of Gut Microbiota in TMAO Metabolism

It has been demonstrated in gnotobiotic mice that gut bacteria are essential to transform the dietary compounds into TMA [18]. Moreover, some studies performed with antibiotics in rats and humans reveal that the production of TMA and TMAO is almost totally suppressed with the use of broad-spectrum antibiotics, such as Ciprofloxacin, Vancomycin or Metronidazole. However, the levels of TMAO return to normal after one month of the withdrawal of the antibiotics [7,52].

Although most of TMA production takes place in the gut, it can also occur in the mouth through the action of some bacteria like *Streptococcus sanguis*. Some studies revealed that the genes *CutC* and *CutD*, which are present in the cluster *Cut*, are indispensable for *Desulfovibrio alaskensis* and *Desulfovibrio desulfuricans* to transform choline into TMA [18]. Moreover, in some bacteria of the gender *Acinetobacter* and *Serratia*, *CntA* and *CntB* gens encode the two subunits of the oxidoreductase enzyme necessary to convert L-carnitine into TMA [18]. On the other hand, *YeaW*/*YeaX* are another gene pair that encode some oxygenase and oxidoreductase enzymes with substrate promiscuity for choline, betaine, carnitine and GBB [18]. These genes, in addition to the orthologs and homologs of the *CntA*/*CntB* and *YeaW*/*YeaX* gene pairs, can be found in a wide range of bacteria present in the gut. Some of these bacteria are Gammaproteobacteria (*E. coli*, *Citrobacter*, *Klebsiella pneumoniae*, *Providencia*, and *Shigella*), Betaproteobacteria (*Achromobacter*), Firmicutes (*Sporosarcina*), and Actinobacteria. However, it seems that bacteria belonging to the genera Bacteroidetes are not able to produce TMA from the dietary compounds [18]. In a recent study, the Firmicutes/Bacteroidetes ratio has been used to predict and study TMAO concentrations in plasma [37].

## 3. Relationship between Atherosclerosis, Cardiovascular Disease and TMAO

Gut microbiome has gained much attention because of its possible role as a promoter of chronic diseases, cancer and even neurological disorders. One of the metabolites of the gut microbiome that seems to be involved in the development of atherosclerosis and has recently been linked to inflammation and obesity is TMAO (Table 2).

Nowadays, atherosclerosis is one of the major causes of CVD, so it is plausible to think that, if high plasma TMAO concentrations are related to the development of atherosclerosis, TMAO is therefore related to CVD as well. Indeed, elevated plasma TMAO levels have been found in people at risk of CVD. These patients usually show greater concentrations of the TMAO precursors too. In this context, it is thought that TMAO could serve as a biomarker in humans to predict prevalence of CVD and increased incidence of major adverse cardiovascular events (MACE), such as myocardial infarction, stroke and even death [7,14,52,53,54,55].

High levels of TMAO precursors like choline and betaine also seem to be independently associated with prevalence and poor prognosis in CVD even after adjustment for cardiovascular risk factors [56]. However, not all studies support this association, i.e., Lever et al., (2014) [53] found that high levels of betaine were associated with cardiovascular risk (CVR) only in diabetic patients. Nevertheless, recent studies suggest that these elevated levels are only associated with higher risk of MACE when high TMAO levels are concomitantly present [57].

Moreover, mice fed a Western diet, a risk factor for CVD, have greater plasma TMAO concentrations and develop cardiac dysfunction and heart fibrosis. They also show a bigger expression of pro-inflammatory cytokines, such as tumor necrosis factor-α (TNF-α) and interleukin-1β (IL-1β) and a decrease in the expression of anti-inflammatory cytokines (IL-10). The treatment with 3,3-dimethyl-1-butanol (DMB), that inhibits the choline TMA lyase enzyme, prevented all those outcomes and lowered plasma TMAO levels [8].

Endothelial dysfunction is another pathologic feature that has been related to TMAO. Although the receptor for TMAO has not yet been described, some studies performed on human monocytic THP-1 cells and human umbilical vein endothelial cells (HUVEC) showed that TMAO treatment enhanced monocyte adhesion through the PKC/NF-κB pathway, thus increasing the expression of VCAM-1 [58]. Moreover, studies performed on human aortic endothelial cells (HAECs) and vascular smooth muscle cells revealed enhanced leukocyte adhesion trough the MAPK/NF-κB pathway [59].

Other studies with rats showed increased endothelial dysfunction and vascular inflammation related to high plasma TMAO levels that promoted oxidative stress. The levels were greater in old rats compared to young ones. The overexpression of pro-inflammatory cytokines and superoxide production in addition to the decrease in the expression of eNOS was restored with the use of DMB [32].

In humans, patients with type 2 diabetes and chronic kidney disease have a higher proportion of TMA-producing microbiota and found a positive correlation between TMAO and some biomarkers of inflammation and endothelial dysfunction [60]. Nevertheless, other reports showed a positive correlation between TMAO and ADMA (endothelial dysfunction biomarker) only in patients with HIV and type 2 diabetes, but found no correlation with high sensitivity C-reactive protein (hsCRP), which is an inflammatory biomarker [61].

Trimethylamine N-oxide also appears to modulate lipid homeostasis, which could act as an important risk factor for CVD. Some investigations revealed that TMAO alters cholesterol and sterol metabolism in various compartments, including the bile acid compartment [7,14,15]. In the study performed by Koeth et al., (2013) [14], TMAO lowered the expression of the main bile acid synthetic enzyme (Cyp7a1), which is the rate-limiting step in the catabolism of cholesterol. Some reports of human *CYP7A1* gene variants have shown that a reduction of *CYP7A1* expression is associated with enhanced atherosclerosis [62,63,64]. Supplementation with dietary choline, carnitine or even direct supplementation of TMAO could also promote suppression of reverse cholesterol transport [7,14,15]. Moreover, dietary supplementation with TMAO also reduced the expression of intestinal cholesterol transporters Niemann-Pick C1-like1 (Npc1L1), which transports cholesterol into enterocytes, and Abcg5/8, which transports cholesterol out of them [14].

Additionally, TMAO increases the expression in macrophages of scavenger receptors CD36 and SR-A1, which promote lipid accumulation and foam cell formation [7,17]. CD36 expression and foam cell formation is induced by oxidatively modified low density lipoproteins (ox-LDL). This effect is enhanced by TMAO. MAPK/JNK pathway seems to play a critical role in TMAO-induced atherosclerosis since, when SB230580 (a MAPK inhibitor) and SP600125 (a JNK inhibitor) were used, foam cell formation and CD36 expression were reduced. Foam cell formation was also attenuated by the use of siRNA-mediated knockdown of *CD36* [13].

Although the relationship between CVD risk and plasma TMAO levels appears to be strong, there are still some inconsistences that weaken this association. Some studies have demonstrated that people after bariatric surgery show short- and long-term higher plasma TMAO levels. This result is unexpected as bariatric surgery is known to reduce CVD risk and high TMAO concentrations are thought to increase it [65,66]. As diet and gut microbiota were not recorded or collected in those studies, TMAO levels could be raised as a result of a surgery-induced change in gut microbiome or a greater ingestion of carnitine as it is a TMA precursor and it is often promoted as a weight loss-inducing supplement.

Moreover, several lines of evidence propose that TMAO might play a protective role in CVD [41,67]. Supplementation with L-carnitine seems to improve some features of CVD [68] although it raises plasma TMAO and TMA levels [69], and even TMAO showed positive effects against atherosclerosis in ApoE^−/−^ transgenic mice expressing cholesteryl ester transfer protein CETP [70].

On the other hand, fish and seafood are rich in TMAO and TMA, with marine fish having bigger concentrations than freshwater fish [71]. It has been speculated that, if high TMAO levels are involved in CVD, then eating more fish would increase risk of CVD. Indeed, some studies have shown that high consumption of fish augmented circulating TMAO levels within 15 min of food consumption, suggesting that TMAO itself can be absorbed without undergoing any transformation by gut microbiota [72]. Nevertheless, the risk of CVD is not increased as fish also contains cardioprotective molecules such us ω3-poly unsaturated fatty acids, mainly eicosapentaenoic acid (EPA) and docosahexaenoic acid (DHA) [73,74]. In fact, the use of fish oil (FO) in mice fed a HFD ameliorated the adverse effects produced by TMAO, including impaired glucose tolerance and adipose tissue inflammation, decreasing MCP-1 and increasing IL-10 levels [36].

In the light of all this evidence, currently it is not totally agreed that TMAO by itself is a proatherogenic compound or just a biomarker of CVD. Several studies point to FMO3 to play an important role in CVD independently of TMAO. In this line, downregulation or knock-out of FMO3 leads to decreased levels of TMAO and it is athero-protective in these models, whereas increased expression of FMO3 leads to pro-atherogenic lipid profiles (e.g., higher VLDL and LDL) [75,76]. Additionally, upregulation of FMO3 may be pro-atherogenic or pro-diabetic via mechanisms unrelated to increased production of TMAO. Increases in FMO3 have been linked to alterations of reverse cholesterol transport [75], and downregulation of FMO3 can prevent hyperglycemia, hyperlipidemia, and atherosclerosis in a mouse model of insulin resistance [76]. In that study, Miao et al. found that FMO3 is required for expression of FoxO1, a key node within the cell, controlling growth, differentiation and metabolism. Moreover, a clear sexual dimorphism in FMO3 has been described. Thus, in female mice, FMO3 is not strongly induced by insulin resistance and non-diabetic female mice have high levels of FMO3/TMAO, but do not necessarily involve hyperglycaemia or atherosclerosis. In male mice, FMO3 is strongly induced by diabetes, but transient overexpression of FMO3 in lean male mice does not produce hyperglycaemia. In both male and female mice, FMO3 is required for the development of the diabetic phenotype. Taken together, these data suggest that FMO3, which is differentially regulated in male and female mice, is necessary but apparently not sufficient for the development of the diabetic phenotype.

## 4. Relationship between TMAO and Neurological Disorders

The gut-brain axis has recently started to gain importance. There seems to be a connection between the gut microbiome and some neurological disorders (Table 3). A recent study evidenced that TMAO can be detected in human cerebrospinal fluid [30]. However, it is not known if the TMAO detected comes from hepatic synthesis and crosses the blood brain barrier (as showed in Vernetti L et al., 2017) [77] or if it comes from de novo synthesis in the brain since FMO3 has been detected in the adult brain [30]. Additionally, TMAO has been suggested to cause blood brain barrier disruption by reducing the expression of tight junction proteins, such as claudin-5 and tight junction protein-1 (ZO-1), favouring its access to the brain [78].

Xu and Wang (2016) [79] used several validated algorithms to identify TMAO as a metabolite significantly associated with various aspects of Alzheimer’s disease (AD). They also found common genetic pathways underlying AD biomarkers and TMAO. Another study [80] demonstrated that TMAO is able to stabilize and modify the aggregation of the amyloid beta (Aβ) peptide, favouring and accelerating the transformation of the random string of the Aβ peptide to its β-conformation and stabilizing the resulting protofibrils, which can originate fibers that tend to aggregate and form plaques. These results suggest that TMAO could help to accelerate and develop better models of Aβ aggregation.

However, these findings contrast with the protective effect that TMAO could exert. Indeed, some studies found out that TMAO and betaine can act as natural osmolytes and stimulate tau-induced tubulin assembly [81]. Moreover, later studies showed the ability of TMAO to promote and enhance the assembly of microtubules in mutant and hyperphosphorylated tau protein, reaching in the majority of cases a greater protein efficiency ratio than in wild-type tau [82]. TMAO seems not to act by dephosphorylating tau protein; it facilitates the binding between tau protein and tubulin by reducing the critical concentration of tubulin necessary for assembly [83].

Organic osmolytes like TMAO could also exert protective effects in prion diseases. For example, TMAO inhibits the conversion of the scrapie prion protein (PrPC) into its pathogenic isoform (PrPSc), which is associated with transmissible spongiform encephalopathies. TMAO seems not to affect the current population of PrPSc, but interferes with the formation of PrPSc from newly sinthethized PrPC [84].

The protective effect exerted by TMAO as a chemical chaperone has also been observed in other diseases, such as Machado-Joseph disease/spinocerebellar ataxia-3 (MJD/SCA-3) [85] and amyotrophic lateral sclerosis (ALS) [86]. On one hand, MJD/SCA-3 is an inherited neurodegenerative disorder where the truncated form of mutated ataxin-3 causes aggregation and cell death in vitro and in vivo. TMAO was found to reduce aggregate formation, cell death and cytotoxicity induced by truncated expanded ataxin-3 [85]. On the other hand, TMAO also seems to be beneficial in ALS. However, the major limitation in using chemical chaperones like TMAO as drugs is their very active concentration, in the millimolar range. Because of that, Getter et al. [86] designed a lipophilic derivative of TMAO, which was able to improve neurological functions in mice, preventing ER-stress-induced apoptosis of NSC-34 motor neuron-like cells and primary mouse astrocytes.

Finally, TMAO has been also shown to play a role in the conformation of intrinsically disordered proteins (IDP). IDP are proteins that do not have a stable three-dimensional structure. One of these IDP is α-synuclein, which can aggregate into toxic protofibrils, is one of the main components of Lewy bodies and has been linked to Parkinson disease (PD) and other neurodegenerative diseases [87]. TMAO suppresses the formation of extended conformations and can act as a protecting osmolyte leading to compact and folded forms of α-synuclein. This effect could probably prevent the alpha-synuclein aggregation and formation of insoluble fibrils that cause PD [83]. When the concentration of TMAO is high enough, α-synuclein forms oligomers in which the subunits are folded and are not able to fibrillate [88].

## 5. Inflammation as the Underlying Mechanism of the Deleterious Effects of TMAO

Several studies have revealed an increase in the expression of pro-inflammatory cytokines when plasma TMAO levels are elevated (Table 4). The study performed by Rohrmann et al. [10] described a link between low grade inflammation and plasma TMAO levels. When the concentration of plasma TMAO was augmented there was an overexpression of TNF-α, IL-6 and C-reactive protein.

The relationship between TMAO and inflammation seems pretty clear. For example, studies performed in fetal human colon cells (FHCs) found a dose- and time-dependent increase of oxidative stress when TMAO was added. Furthermore, they also observed a dose-dependent inhibition of the expression of ATG16L1, decreasing ATG16L1-induced autophagy and activating NLRP3 inflammasome, which has been recently found to be critical for the development of atherosclerosis and has been also linked to AD [89,90]. Those harmful effects were significantly reversed by siRNA-mediated knockdown of *NLRP3* and over-expression of *ATG16L1* [12].

Other experiments performed in HUVEC and mice also revealed a link between TMAO administration and inflammation by activation of NLRP3 inflammasome. One of these studies suggests that this activation occurs through the inhibition of SIRT3-SOD2-mitochondrial ROS signalling pathway. Additionally, TMAO could not further inhibit *Sod2* in SIRT3 siRNA-treated HUVEC and aortas from SIRT3^−/−^ mice [9]. Other studies propose that this activation is mediated through ROS-TXNIP pathway, elevating inflammatory cytokines, IL-1β and IL-18 and inhibiting endothelial nitric oxide synthase (eNOS), thus decreasing the production of nitric oxide (NO). These effects can be reversed with the use of N-acetylcysteine (NAC) or siRNA-mediated knockdown of *TXNIP* and *NLRP3* [11].

Moreover, some trials performed in carotid artery endothelial cells (CAEC) revealed that TMAO significantly increases the activation and formation of NLRP3, caspase-1 activity, IL-1β production and cell permeability. This activation of NLRP3 was abolished with NLRP3 siRNA or caspase-1 inhibitor, WEHD [89].

## 6. Therapeutic Strategies

The discovery of TMAO as a metabolite produced by gut microbiota and metabolized in the liver that plays a role in systemic inflammation, atherosclerosis and vascular dysfunction, has raised the possibility of treating these diseases by targeting gut microbiota and their metabolites (Table 5).

The use of prebiotics and probiotics could be useful to elicit a favourable impact on gut microbiota composition. Prebiotics include all types of non-digestible foods, such as oligosaccharides, that stimulate the growth of beneficial bacteria [26], while probiotics include the administration of specific bacterial strains. Those bioactive foods could be useful to decrease bacteria able to transform precursors into TMA and to increase bacteria able to deplete it or bacteria without the genes needed to convert carnitine or choline into TMA. As an example, although it has to be demonstrated in humans, the administration of *Lactobacillus paracasei* in mice expressing human baby microbiota reduced TMA production [91]. Other studies have proposed the use of methanogenic bacteria to deplete TMA and TMAO [92,93]. A large number of bacteria belonging to the order Methanobacteriales have been found in the human gut. These Archaea use methyl compounds such as TMA and TMAO as substrate to generate methane [22,31].

Instead of changing the gut microbiota, the option of reducing carnitine or choline levels in the diet would not be a possibility because they are important nutrients and low levels of them can lead to organ dysfunction. Indeed, some of the main functions of choline and its derivatives are the production of neurotransmitters (like acetylcholine) and the stability of the cell membrane (as phosphatidylcholine). Furthermore, L-carnitine helps to maintain skeletal and cardiac muscle function and may be useful in the reduction of MACE and mortality after acute myocardial infarction [94].

The use of antibiotics as a therapy to eliminate microbiota able to transform dietary precursors (choline, betaine and L-carnitine) into TMA has also been devised. The use of broad spectrum antibiotics such us ciprofloxacin and metronidazole leads to near almost complete suppression of TMAO levels. However, one month after the withdrawal of antibiotics, TMAO levels are detectable again [52]. Other studies performed in mice using a mix of vancomycin, neomycin-sulphate, metronidazole and ampicillin, showed an inhibition of dietary choline-enhanced atherosclerosis. Plasma TMAO levels were supressed and macrophage foam cell formation was inhibited [7]. Albeit the use of antibiotics is effective in the suppression of microbiota that produces TMA, the chronic use of antibiotics is not viable since it can lead to resistant bacterial strains and repopulation. Moreover, antibiotics do not only kill harmful bacteria, but could affect the beneficial too [52].

Another possibility could be the use of oral non-absorbent binders to remove TMAO or its precursors. For example, oral charcoal adsorbent (AST-120) has been clinically used to remove uremic toxins such as indoxyl sulfate from patients with advanced renal failure. However, a compound that removes specifically TMAO has not yet been discovered [18,95].

Other proposed therapeutic approaches include the use of some analogues or the inhibition of TMA precursors. In the study performed by Hui et al. [37], the inhibition of the phospholipase-autotaxin pathway, which generates choline and lysophosphatidic acid from lysophospholipids such as lysophosphatidylcholine, was suggested. Other studies propose the use of DMB, an analogue of choline that inhibits choline TMA lyase. DMB can be found in some foods like balsamic vinegars, red wines or extra-virgin olive oil and grapeseed oil [96]. Although it inhibits the transformation of choline, carnitine and crotonobetaine into TMA in mice and rats, DMB is not able to avoid the complete TMAO synthesis as it cannot inhibit the conversion of GBB to TMA [8,17,32,38]. There are other compounds such as meldonium that has been also studied. Meldonium is an aza-analogue of GBB with cardioprotective effects that is used as an anti-ischemic and anti-atherosclerotic drug. Moreover, it seems to reduce plasma TMAO levels in humans by increasing its urinary excretion and reducing its biosynthesis from L-carnitine (inhibiting the conversion of GBB into L-carnitine) [97]. Contrarily, other studies performed in rats suggest that meldonium decreases the excretion and production of TMAO. It increases GBB levels and reduces TMA formation from L-carnitine, but not from choline [98].

Another appealing approach is the inhibition of the enzymes involved in TMA biosynthesis. The gene cluster responsible for the conversion of choline into TMA has been identified. *CutC* and *CutD* are necessary for that transformation; so they could be used like targets as their inhibition eradicates the production of TMA [18]. The inhibition of the enzymes able to generate TMAO could be another option. The knockout of *mo3* normalizes TMAO levels in mice [76]. Nevertheless, there could be several problems with this approach as an accumulation of TMA leads to trimethylaminuria, which is characterized by fishy odor (similar to rotten fish) and induce inflammation [18,75,99]. Another problem would be that TMAO is not the only substrate for FMO3; morphine, propranolol and tyramine are also metabolized by FMO3 [18].

Several studies have associated the use of some herbal products with lower plasma TMAO levels. For example, the use of *Gynostemma pentaphyllum* (a plant used in China to treat hyperlipidemias and obesity) seems to reduce plasma TMAO levels and increase lecithin levels in rats [39]. On the other hand, the use of Gancao (the root of *Glycyrrhiza uralensis*) seems to reduce TMAO levels when Fuzi (the processed lateral root of *Aconitum carmichaelii*) is co-administered [100]. Other compounds such as resveratrol could modulate gut composition, decreasing TMA-forming bacteria and increasing the amount of *Lactobacillus* and *Bifidobacterium*. Thus, plasma TMAO levels decrease. Resveratrol did not show beneficial effects when antibiotics were used concomitantly [101]. These studies were performed in mice, but similar results could be expected in humans.

Finally, the administration of enalapril has recently been linked to lower plasma TMAO levels in rats, probably by increasing urine TMAO excretion. However, the mechanism is uncertain as enalapril did not achieve to decrease indoxyl sulphate levels [102]. This study also demonstrated that enalapril does not affect TMA production or gut bacteria composition.

## 7. Concluding Remarks

Recent studies point to the potential contribution of gut microbiota-derived production of TMAO from the metabolism of dietary choline and L-carnitine, which has been associated with an increased risk of major adverse events in humans. Indeed, several experimental studies suggest a possible involvement of TMAO in the etiology of CVD and neurological disorders. Interestingly, the close link between TMAO and inflammatory stages suggests that inflammation could underlie some of the deleterious effects of TMAO. Therefore, it is important to undertake studies examining intracellular concentrations of TMAO in mammals, cellular signaling and also determine the effects of TMAO on enzymes and other proteins in order to establish the role of TMAO and to study the potential utility of TMAO as an early biomarker or a target for the prevention of disease in humans.

## Figures and Tables

**Table 1:** Effects of different types of diet on gut microbiota and TMAO levels in different experimental models and metabolic circumstances.

Type of diet	Influence on microbiota and TMAO	Consequences and Remarks	References
**High fat diet (mice)**	↑ Plasma TMAO	Obesity and metabolic problems (not prevented with the use of DMB) Renal fibrosis, oxidative stress and inflammation of the kidney (prevented with DMB).	Sun G et al., 2017
**High fat diet enriched in phosphatydilcholine (rats)**	↑ TMAO in plasma and liver	Hyperlipidemia. TMAO levels decrease and lecithin levels increase with treatment of *Gynostemma pentaphyllum*, but not with treatment of atorvastatin.	Wang M et al., 2013
**High fat diet (human)**	↑ Postpandrial plasma TMAO levels, but not fasting ones.	In the short term, a reduction of plasma TMAO clearance is observed.	Boutagy NE et al., 2015a
**High fat diet (human)**	↑ Plasma TMAO	The increase of TMAO levels is not prevented with the use of probiotics (VSL#3^®^), though there is less weight gain and fat. The magnitude of the change in the levels of TMAO is correlated with systolic pressure and carotid pulse.	Boutagy NE et al., 2015b
**High fat diet**	↑ Firmicutes and Proteobacteria ↓ Bacteroidetes	More production of TMAO.	Hui D, 2016
**Low fat diet**	↑ Bacteroidetes and ↓ Firmicutes	Lower production of TMAO.	Hui D, 2016
**High fat diet enriched in fish oils (FO) (mice)**	↑ TMAO plasma	FO improve the adverse effects produced by TMAO (tolerance to glucose and adipose tissue inflammation).	Gao X et al., 2015
**High protein diet**	↑ TMAO urine	High correlation with daily nitrogen excreted through the urinary tract.	Rasmussen LG et al., 2012
**Low protein diet**	↓ TMAO plasma	A diet low in proteins in patients with CKD resulted in lower plasma TMAO levels.	Mafra D et al., 2017
**Similar to Western (mice)**	↑ TMAO plasma	Obesity and Dyslipidemia (not prevented with the use of DMB) Cardiac dysfunction and fibrosis of heart with increased expression of Pro-Inflammatory Cytokines, tumor necrosis factor and interleukin IL-1β and reduced expression of anti-inflammatory cytokines (IL-10) (prevented with DMB).	Chen K et al., 2017
**Rich in indigestible CH (human)**	↓ Production of TMAO Gut microbiota alterations	Significant weight loss in children with simple obesity or Prader Willi Syndrome (PWS). Better state of inflammation.	Zhang C et al., 2015
**Rich in CH and/or low in CH and rich in indigestible starch (human)**	↑ TMAO plasma	The diet does not improve short-term biomarkers of CVR. It mitigates the postprandial glucose and insulin response to hearty meals.	Bergeron N et al., 2016
**Supplementation with pistachios (human)**	↓ Production of TMAO	Improvement of metabolic disorders associated with IR and DMII.	Hernández-Alonso P et al., 2017
**Supplementation with histidine (human)**	↑ TMAO in plasma and urine.	Lower production of lipids and glucose.	Du et al., 2017
**Vegetarian (human)**	Changes in gut microbiota	Vegetables of the family Cruciferae can reduce FMO3 activity Reduced ability to produce TMA from L-carnitine.	Koeth RA et al., 2013

CH: Carbohydrates; CKD: Chronic kidney disease; CVR: Cardiovascular risk; DMB: 3,3-Dimethyl-1-butanol; DMII: Diabetes mellitus type II; FMO3: Flavin-dependent monooxygenase isoform 3; FO: Fish oil; IR: Insulin resistance; TMA: Trimethylamine; TMAO: Trimethylamine N-oxide; ↑: increase; ↓: decrease.

**Table 2:** Relationships between atherosclerosis, cardiovascular disease and TMAO in different experimental models.

Species/Cells	Alterations of TMAO levels, consequences and remarks // Proposed Mechanisms	References
THP-1 y HUVECs (Human Umbilical Vein Endothelial Cells)	↑ TMAO levels relates to: ↑ Endothelial dysfunction ↓ Endothelial self-reparation ↑ Adhesion of monocytes through activation of: PKC/NF-κB/VCAM-1 pathways	Ma G et al., 2017
Mouse, HAECs (Human Aoric Endothelial Cells) and VSMC (Vascular Smooth Muscle Cells)	↑ TMAO levels relates to: ↑ Proinflammatory cytokines via MAPK and NF-κB ↑ Leukocyte adhesion to endothelial wall	Seldin MM et al., 2016
Mouse	↑ TMAO levels relates to: ↑ Proinflammatory cytokines ↑ Tumor necrosis factor ↑ Interleukin IL-1β ↓ Anti-inflammatory cytokines (IL-10)	Chen K et al., 2017
Human	↑ TMAO levels relates to prevalent CVD	Wang Z et al., 2011 Mente A et al., 2015
Human	Higher TMAO levels in patients after bariatric surgery	Troseid M et al., 2016
Human	Higher TMAO levels in patients after bariatric surgery in the short and long term	Narath S et al., 2016
Human and mice	TMAO alters cholesterol and sterol metabolism in various compartment TMAO lowers the expression of the main bile acid synthetic enzyme (Cyp7a1) Supplementation of TMAO precursors or even TMAO itself, could also promote suppression of reverse cholesterol transport TMAO also reduces the expression of intestinal cholesterol transporters Niemann-Pick C1-like1 (Npc1L1)	Koeth RA et al., 2013; 2014 Wang Z et al., 2011
Mice	TMAO increases the expression in macrophages of scavenger receptors CD36 and SR-A1, which promote lipid accumulation and foam cell formation	Wang Z et al 2011, 2015
Mice	TMAO enhanced CD36 expression and foam cell formation, which is induced by oxidatively modified low density lipoprotein (ox-LDL). Foam cell formation was also attenuated by the use of siRNA-mediated knockdown of CD36	Geng J et al., 2018
Rat	Circulating TMAO levels increase with age ↑ Endothelial dysfunction and vascular inflammation via oxidative stress ↑ Expression of pro-inflammatory cytokines ↓ eNOS expression (corrected with DMB)	Li T et al., 2017
Human	Patients with T2D and chronic kidney disease have more amount of TMAO producing microbiota. There is a positive correlation with endothelial dysfunction and inflammatory biomarkers	Al-Obaide MAI et al., 2017
Human	TMAO is correlated with ADMA (marker of endothelial dysfunction) in patients with DMII and HIV, not in the other groups. Uncorrelated to hsCRP	Hove-Skovsgaard et al., 2017
Human	High levels of betaine were associated with CVR only in diabetic patients	Lever M et al., 2014
Human	Elevated levels of TMAO precursors are only associated with higher risk of MACE when high TMAO levels are present concomitantly	Wang Z et al., 2014b
Human	Supplementation with L-carnitine seems to improve some features of CVD although it raises plasma TMAO and TMA levels	Fukami K et al., 2015
Mice	TMAO shows positive effects against atherosclerosis in ApoE-/-transgenic mice expressing cholesteryl ester transfer protein CETP	Collins HL et al., 2016
Human	↑ TMAO levels relates to: ↑ Cardiac failure ↓ survival Diastolic dysfunction Uncorrelated with markers of inflammation	Tang WH et al., 2015b
Murine macrophage J774A.1 cells	↑ TMAO levels relates to: ↑ Expression of SR-A1 (proatherogenic), ↑ Stress in endoplasmic reticulum ↓ ATP-binding cassette transporter A1	Mohammadi et al., 2016

DMB: 3,3-dimethyl-1-butanol; ADMA: Asymmetric dimethylarginine; DMII: Type 2 diabetes mellitus; CVR: Cardiovascular risk; MACE: Major adverse cardiac events; CVD: Cardiovascular disease; TMAO: Trimethylamine N-oxide; TMA: Trimethylamine; IL: Interleukin; eNOS: Endothelial nitric oxide synthase; DMB: 3,3-Dimethyl-1-butanol; T2D: Type 2 diabetes; HIV: Human immunodeficiency virus; SR-A1: Class A1 scavenger receptors; ↑: increase; ↓: decrease.

**Table 3:** Relationships between neurodegenerative disorders and TMAO in different experimental conditions.

Experimental conditions	Remarks	References
Human	Detection of TMAO in cerebrospinal fluid. It seems that TMAO levels are not related to neurological disorders (although it was not the objective of the study).	Del Rio D et al., 2017
Synthesized and purified Aβ peptides	TMAO is able to stabilize and modify the aggregation of the peptide Aβ, favouring and accelerating the transformation of the random string of the Aβ peptide to its β-conformation and stabilizing the resulting protofibrils, that can originate fibers that tend to aggregate and form tangled plates.	Yang DS Et al., 1999
Wild and mutant tau proteins	TMAO is able to promote and enhance the assembly of microtubules in mutant and hyperphosphorylated tau protein, reaching in the majority of cases a greater protein efficiency ratio than in wild-type tau.	Smith MJ Et al., 2000
Purified human recombinant tau	TMAO does not act by dephosphorylating tau protein; it facilitates the binding between tau protein and tubulin by reducing the critical concentration of tubulin necessary for assembly.	Tseng HC et al., 1999
Human	TMAO has been suggested to cause blood brain barrier disruption by reducing the expression of tight junction proteins like claudin-5 and tight junction protein-1 (ZO-1).	Subramaniam S et al., 2018
Purified tau proteins	TMAO can act as a natural osmolyte and stimulates tau-induced tubulin assembly	Tseng HC and Graves DJ, 1998
Scrapie-infected mouse neuroblastoma cells	TMAO inhibits the conversion of the scrapie prion protein (PrPC) into its pathogenic isoform (PrPSc), which is associated with transmissible spongiform encephalopathies.	Tatzelt J et al., 1996
BHK-21 and Neuro2a cells transfected with N-terminal truncated ataxin-3 with an expanded polyglutamine stretch	TMAO has been shown to reduce aggregate formation, cell death and cytotoxicity induced by truncated expanded ataxin-3, which is involved in Machado-Joseph disease/spinocerebellar ataxia-3.	Yoshida H et al., 2002
Mice	A lipophilic derivative of TMAO showed an improvement in neurological functions in mice, preventing endothelial reticulum-stress induced apoptosis of NSC-34 motor neuron-like cells and primary mouse astrocytes.	Getter T et al., 2015
α-synuclein peptides	TMAO suppresses the formation of extended conformations and can act as a protecting osmolyte leading to compact and folded forms of α-synuclein. This effect could probably prevent the alpha-synuclein aggregation and formation of insoluble fibrils that cause Parkinson disease.	Jamal S et al., 2017
Purified recombinantα –synuclein	When the concentration of TMAO is high enough, α-synuclein forms oligomers in which the subunits are folded and are not able to fibrillate.	Uversky V et al., 2001

TMAO: Trimethylamine N-oxide.; Aβ: Amyloid beta; ZO-1: Tight junction protein-1; PrPC: Scrapie prion protein.

**Table 4:** Relationships between inflammation and TMAO in different in vivo and cellular systems.

Species // Cell lines	Alterations of TMAO levels, consequences and remarks // Proposed mechanisms	References
Human	↑ PlasmaTMAO levels relates to: ↑ TNF-α, ↑ IL-6 ↑ C-reactive protein ↑ Inflammation	Rohrmann S et al., 2016
Fetal human colon cells (FHC)	TMAO increases state inflammation via NLRP3 inflammasome activation (gets reversed with ATG16L1 overexpression or siRNA-NLRP3 KO) ↑ TMAO levels inhibit cell growth and ↑ apoptosis. It also induces oxidative stress and inhibits the expression of ATG16L1 and p62 and the autophagy of LC3-II	Yue C et al., 2017
Human umbilical vein endothelial cells (HUVEC)	TMAO increases oxidative stress and inflammation via ROS-TXNIP-inflammasome NLRP3. It also increases IL-1β and IL-18 and inhibits eNOS and NO. The effects are reversed with the use of NAC and siRNA-mediated knockdown TXNIP - NLRP3	Sun X et al., 2016
Mouse and HUVEC	TMAO promotes vascular inflammation by activating the NLRP3 inflammasome ↑ IL-1β, ICAM-1 and MMP-9 ↑ Monocyte adhesion to endothelial cells NLRP3 activation is mediated by inhibition of SIRT3-SOD2-mtROS signalling pathway	Chen ML et al., 2017
Carotid artery endothelial cells (CAEC)	TMAO significantly increases the activation and formation of NLRP3 and caspase-1 activity, ↑ IL-1β production and cell permeability Activation of NLRP3 was abolished with NLRP3 siRNA or caspase-1 inhibitor, WEHD	Boini KM et al., 2017

TMAO: Trimethylamine N-oxide; TNF-α: Tumor necrosis factor α; FHC: Fetal human colon cells; HUVEC: Human umbilical vein endothelial cells; IL: Interleukin; ICAM-1: Intercellular Adhesion Molecule 1; MMP-9: Matrix metallopeptidase 9; ↑: increase; ↓: decrease.

**Table 5:** Proposed therapeutic strategies targeting TMA metabolism.

Therapy	Effects	Remarks and Issues
Prebiotics	Elicit a favourable impact on gut microbiota composition to decrease TMA formation in the intestine.	Unclear effects in humans. Several factors influence gut microbiota composition
Probiotics (I): Bacteria unable to transform precursors into TMA	Decrease TMA formation in the gut	Beneficial effects in mice. However, the effects are not clear in humans
Probiotics (II): Methanogenic bacteria	Deplete TMA and TMAO	Safety and engraftment remain unclear in humans
Antibiotics	Eliminate TMA-forming microbiota. Nearly total suppression of plasma TMAO levels	Nonspecific, beneficial bacteria are also eradicated. Chronic use is not viable. Repopulation and resistant bacterial strains are likely
Oral non-absorbent binders	Remove TMAO or its precursors in the gut	Hypothetical approach. A compound that removes specifically TMAO has not yet been discovered
FMO3 enzyme inhibition	Prevents TMA oxidization to TMAO	An accumulation of TMA produces trimethylaminuria, characterized by fishy odor, and could cause inflammation. FMO3 also metabolizes other molecules
*Gynostemma pentaphyllum*	Reduces plasma TMAO levels and increase lecithin levels	The effect of this plant in the other precursors has not been studied. Studies performed in rats
Gancao	Avoids the increase in TMAO levels when Fuzi is co-administered	It does not reduce TMAO levels when administered alone. Studies performed in rats
Resveratrol	Modulates gut microbiota composition. ↓ TMA and TMAO production	↑ *Lactobacillus* and *Bifidobacterium* No effects when antibiotic are used. Studies performed in mice
3,3-Dimethyldimethyl-1-butanol (DMB)	Inhibits transformation of choline, carnitine and crotonobetaine into TMA through inhibition of microbial TMA lyases	Not able to inhibit the conversion of γ-butyrobetaine to TMA. Studies performed in mice and rats
Meldonium	Reduces TMAO biosynthesis from L-carnitine (inhibits the conversion of GBB into L-carnitine)	Not able to reduce TMAO formation from choline. It may increase TMAO urinary excretion in humans
Enalapril	Increases urine TMAO excretion	Unknown mechanism. Studies performed in rats. It does not affect TMA production or gut bacteria composition

TMAO: Trimethylamine N-oxide; TMA: Trimethylamine; ↑: increase; ↓: decrease.
